# Deep Neural Network-Based Fusion Localization Using Smartphones

**DOI:** 10.3390/s23218680

**Published:** 2023-10-24

**Authors:** Suqing Yan, Yalan Su, Jianming Xiao, Xiaonan Luo, Yuanfa Ji, Kamarul Hawari Bin Ghazali

**Affiliations:** 1 Guangxi Key Laboratory of Precision Navigation Technology and Application, Guilin University of Electronic Technology, Guilin 541004, China; yansuqing@guet.edu.cn; 2 School of Information and Communication, Guilin University of Electronic Technology, Guilin 541004, China; annsue174@gmail.com; 3 Department of Science and Engineering, Guilin University, Guilin 541006, China; 4 Guangxi Key Laboratory of Image and Graphic Intelligent Processing, Guilin University of Electronic Technology, Guilin 541004, China; luoxn@guet.edu.cn; 5 National & Local Joint Engineering Research Center of Satellite Navigation Localization and Location Service, Guilin 541004, China; jiyuanfa@guet.edu.cn; 6 GUET-Nanning E-Tech Research Institute Co., Ltd., Nanning 530031, China; 7 Faculty of Electrical and Electronics Engineering, Universiti Malaysia Pahang, Pekan 25200, Malaysia; kamarul@ump.edu.my

**Keywords:** indoor localization, deep neural networks, magnetic, dead reckoning, step length estimation, smartphone

## Abstract

Indoor location-based services (LBS) have tremendous practical and social value in intelligent life due to the pervasiveness of smartphones. The magnetic field-based localization method has been an interesting research hotspot because of its temporal stability, ubiquitousness, infrastructure-free nature, and good compatibility with smartphones. However, utilizing discrete magnetic signals may result in ambiguous localization features caused by random noise and similar magnetic signals in complex symmetric and large-scale indoor environments. To address this issue, we propose a deep neural network-based fusion indoor localization system that integrates magnetic and pedestrian dead reckoning (PDR). In this system, we first propose a ResNet-GRU-LSTM neural network model to achieve magnetic localization more accurately. Afterward, we put forward a multifeatured-driven step length estimation. A hierarchy GRU (H-GRU) neural network model is proposed, and a multidimensional dataset using acceleration and a gyroscope is constructed to extract more valid characteristics. Finally, more reliable and accurate pedestrian localization can be achieved under the particle filter framework. Experiments were conducted at two trial sites with two pedestrians and four smartphones. Results demonstrate that the proposed system achieves better accuracy and robustness than other traditional localization algorithms. Moreover, the proposed system exhibits good generality and practicality in real-time localization with low cost and low computational complexity.

## 1. Introduction

Since the tremendous development of microelectromechanical systems (MEMS), smartphones with built-in sensors have become an important measuring instrument for Location-based services (LBS) [1]. Typical applications for location-based activities related to people’s daily lives, such as pedestrian and target navigation, smart advertising, and location-based posts, are all related to smartphones. With a built-in global navigation satellite system (GNSS) chip [2], accurate real-time localization can be achieved in open-sky outdoor environments. However, the satellite signals are easily blocked by the building structure or other obstructions, resulting in inferior navigation in indoor environments. Nowadays, campuses, shopping malls, and other interior buildings are becoming increasingly complex [3]. Therefore, practical applications like location tracking of children and elderly people inside buildings, tourist route design experiences in museums, location-based social networking, and intelligent delivery services urgently need the support of high-precision indoor LBS with smartphones.

Smartphones integrated with inertial measurement unit (IMU) sensors, such as accelerometers, gyroscopes, and magnetometers, can provide enough information for pedestrian location [1]. Magnetic fingerprint-based localization [4,5] and Pedestrian dead-reckoning (PDR) localization [6,7,8] are navigation technologies capable of using smartphone sensors to achieve indoor localization without extra infrastructure. Magnetic fingerprint-based localization can overcome the cumulative and drift errors of long-term localization and does not depend on the initial position, making it capable of independent localization. PDR localization, on the other hand, has all these limitations and is thus usually combined with other methods. The integrated method fusing magnetic and PDR is a research hotspot to achieve high-precision and real-time localization using smartphones in indoor environments [9,10]. Wang et al. [11] designed a walking pattern-based PDR algorithm, and the magnetic fingerprint was utilized to calibrate the trajectory. Li et al. [12] proposed a fusion localization system aided by magnetic matching, using an augmented particle filter to integrate it with PDR to estimate the pedestrian location.

The temporal stability and ubiquity of the magnetic signals make magnetic fingerprint-based localization available. However, the utilization of discrete magnetic signals may lead to location feature ambiguity due to random noise and similar magnetic signals. Therefore, the critical factor of magnetic fingerprint-based localization is to identify more unique characteristics associated with location information in magnetic sequences with restricted dynamic range, thereby achieving stable and reliable localization results [13]. Indoor localization using magnetic time series sequences is considered to learn the relationship between temporal-correlated location labels and magnetic sequences. A representative algorithm is the dynamic time warping (DTW) algorithm [4,14,15,16,17], which obtains the target location by aligning and calculating the similarity between the magnetic signals and the fingerprint database. Chen J. et al. [18] put forward 3-dimensional dynamic time warping (3DDTW) to estimate the similarity between the magnetic sequences and the fingerprint database. Chen L.N. et al. [19] proposed a localization system called Meshmap, which uses DTW to achieve magnetic matching and location correction. However, since the DTW algorithm performs global matching in the fingerprint database, the localization error and computational complexity are significantly large. Particle filtering is another typical magnetic sequence-based algorithm [20,21]. Viset et al. [22] proposed a simultaneous localization and mapping (SLAM) method for magnetic ambient and PDR localization using an extended Kalman filter. Shi et al. [23] first put forward a magnetic fingerprint dataset construction method in 2-D, then proposed an integrated PF algorithm to improve PDR instability. Good performance can be obtained by using particle filters for magnetic localization, but particle degradation is still an unavoidable problem for particle filters. The magnetic length also affects localization accuracy and computational complexity. In addition, a large fingerprint database will cause overmatching, which increases the probability of localization ambiguity. 

IMU sensors embedded in smartphones can assist in PDR localization by estimating pedestrian walking characteristics in indoor environments, including step detection, step length, and heading angle estimation. PDR has relative localization accuracy over a short distance and has been combined with other methods. Yan et al. [24] devised a fusion localization method using inertial sensors and acoustic signals. An adaptive step length estimation was proposed to improve PDR localization. In Ref. [25], it also designed an acoustic and PDR-based localization system. The PDR localization was improved by a step length estimation and a heading direction estimation. Tong et al. [26] presented a hybrid localization method integrated with PDR and UWB to improve localization accuracy. The results demonstrated that the algorithm performs with better accuracy than the conventional UWB and PDR methods in harsh environments. Tao et al. [27] proposed an indoor localization using PDR and ambient light. A group-weighted averaging algorithm was put forward to estimate the heading direction. Xu et al. [28] devised a real-time localization system combining Bluetooth low energy (BLE) and PDR localization algorithms, which uses an improved robust filter to detect and suppress the errors caused by the BLE localization algorithm. However, some of these methods rely on infrastructure facilities. Other methods may be subject to environmental influences and interference from non-line-of-sight (NLoS) propagation.

To address these challenges, we propose a fusion localization system integrating magnetic and PDR using deep learning. In this system, since PDR has relative precision in the short term, the ambiguous location caused by undistinctive magnetic signals can be detected and effectively eliminated. Meanwhile, magnetic localization can inhibit the cumulative errors of the PDR method over time. Particle filtering is utilized to fuse the magnetic and PDR localization methods. The proposed fusion localization system can sufficiently alleviate the errors caused by magnetic and PDR, thus providing more accurate and trustworthy localization with smartphones. The major contributions of this paper are listed below:

- **A ResNet-GRU-LSTM network model:** In this article, we propose a ResNet-GRU-LSTM neural network model to achieve magnetic localization. A residual network is adopted to improve the magnetic feature extraction ability. Then, the GRU and LSTM are utilized to extract global features. Experiments show that the proposed model can achieve better performance compared to other single-source magnetic algorithms and good robustness among heterogeneous devices.
- **Multifeature-driven step length estimation:** We designed a multifeature-driven step length estimation to achieve accurate step length. Instead of using only acceleration values, we designed an 8-dimensional dataset for more feature extraction. A hierarchy-based GRU neural network is proposed to estimate step length. Experiments demonstrated that the proposed step length estimation can achieve accurate step length and solve the issues of error accumulation and equipment heterogeneity.
- **Fusion localization system framework:** To enhance the localization performance, we propose a fusion localization system in the particle filter framework. We have developed extensive experiments with different pedestrians, scenes, and heterogeneous devices. Experiments validate that comparable localization performance and robustness can be achieved, exhibiting great practicality in real-world applications.

The rest of this paper is arranged as follows: We present the related work in Section 2. Section 3 describes the materials and methodology of the proposed fusion localization system. In Section 4, extensive experiments are conducted, and the evaluation of the experimental results is discussed. Finally, Section 5 presents the conclusion of this paper. 

## 2. Related Work

Indoor localization using deep learning has been widely explored in the navigation field, which can extract high-level abstract features automatically. 

For magnetic localization, a magnetic fingerprint database and corresponding location labels are utilized for neural network training. Once the training is complete, the target location can be predicted. Zhang et al. [29] put forward DeepPositioning, a localization system that integrates geomagnetic and WiFi signals to obtain accurate indoor localization. Liu et al. [30,31] proposed ST-Loc, utilizing a hierarchical bidirectional LSTM network to extract multiple adaptive representations of magnetic sequences for localization. Ashraf et al. [32] presented an indoor localization method called MINLOC, using geomagnetic field patterns and convolutional neural networks (CNN) to determine the pedestrian’s current location. Ouyang et al. [33] proposed a magnetic field localization system using a temporal convolutional network to avoid the vanishing gradient problem. Jin et al. [13] devised a localization algorithm based on the LSTM network to achieve reliable localization results that fully explored the deep features of the magnetic sequence. Sun et al. [34] designed an indoor localization system based on magnetic field data and BLE that uses CNN to classify the floor and location. Bae et al. [35] used a recurrent neural network (RNN) to track the magnetic signals sensed with the object motion for localization. Zhang et al. [36] devised a feature expansion method using the double sliding window for more dimensional features, and then the LSTM network is constructed for robust indoor localization. Shu et al. [37] proposed a deep learning-based magnetic localization utilizing direction-aware multiscale recurrent neural networks (DM-RNNs), which extract various characteristics of magnetic sequences adaptively to achieve precise localization.

Deep learning-based step length estimation has been widely researched in the past few years. Xing et al. [38] presented a backpropagation ANN (BP-ANN) to calculate step length. Wang et al. [39] proposed TapeLine, which utilizes a LSTM module and denoising autoencoders for step length estimation. Ping et al. [40] designed a step length estimation algorithm based on BiLSTM that is able to effectively extract step length features and realize accurate step length estimation under multiple walking conditions. Gu et al. [41] utilized stacked autoencoders and an affine regression layer to achieve step length estimation accurately. Bo et al. [42] put forward a neural network-based step length estimation system called MINN, which utilizes multi-level ResNet and GRU and a multisource unsupervised domain adaptation (UDA) network to extract spatial-temporal and mode-invariant characteristics, respectively. Hannink et al. [43] employed CNN to map inertial sensor data for a specific step to the obtained step length. Zhang et al. [44] proposed an online sequential extreme learning machine to achieve step length. Edel et al. [45] utilized BiLSTM to detect step numbers and a linear model to calculate step length from raw inertial sensor data. Klein et al. [46] proposed the step length estimation algorithm StepNet, consisting of an activity recognition model and a regressor.

Enlightened by the remarkable achievements of deep neural networks in the navigation field, we proposed a fusion indoor localization system based on deep neural networks that uses magnetic and PDR algorithms. A ResNet-GRU-LSTM network model is designed to learn the relationship between magnetic databases and corresponding location labels, thus achieving magnetic localization results. In the PDR algorithm, a hierarchy-based GRU neural network is built to estimate an accurate step length. Finally, we utilize a particle filter to obtain the fused localization, which can provide a more stable and accurate pedestrian location. 

## 3. Materials and Methodology

In this section, we first presented the overview in Section 3.1. Then, the data preprocessing of magnetic and IMU data are illustrated in Section 3.2. In Section 3.3, magnetic localization based on the ResNet-GRU-LSTM network model is introduced. Finally, Section 3.4 illustrates the multifeature-driven step length estimation.

### 3.1. Overview

**Figure 1:**
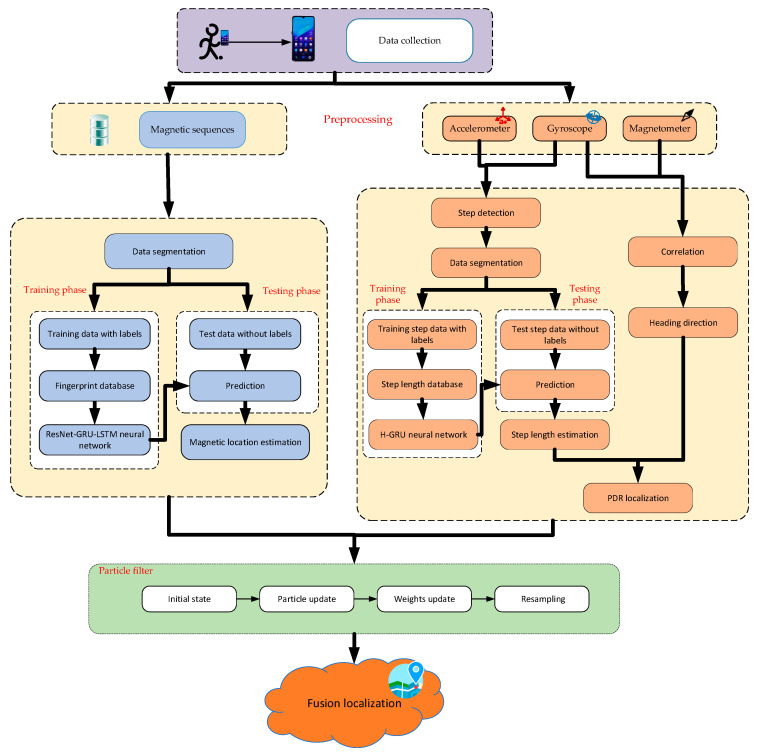
The overall structure of the fusion algorithm localization.

The overall structure of our proposed fusion localization system is illustrated in Figure 1.

In this schematic, a data acquisition application is preinstalled on a smartphone. Then, the pedestrian holds the smartphone while walking along the planned trajectories at a constant speed. The magnetic, acceleration, gyroscope, and orientation data will be collected automatically and stored as *.txt files. These data will be sent to the server terminal for preprocessing. 

In our proposed localization system, both magnetic and PDR-based pedestrian location estimations are achieved by the deep learning method. In magnetic localization, a ResNet-GRU-LSTM network model is proposed to extract unique spatial-temporal features and thus achieve accurate prediction of pedestrian location. In PDR localization, we build a hierarchy GRU neural network for step length estimation; therefore, the accuracy of PDR localization can be improved. In addition, multidimensional datasets of magnetic signals and step length are created for more feature extraction. Finally, the deep learning-based magnetic and PDR localizations are fused through the particle filter framework. 

In the fused localization system, the initial location is determined by magnetic estimation. Then, the PDR estimation is utilized to model the particle motion, thus achieving the particle update. After updating the particle location, the weights of all particles are obtained from magnetic estimation locations. The particles are resampled based on the weight values, and then the location is estimated using these resampled particles. 

The details of the fusion localization system are presented in the following stages.

Stage 1: Initialize the particle. We generate the initial particle set $P_{i}^{t}=\left(locx_{i}^{t},locy_{i}^{t}\right),i=1,\ldots ,N$ at time *t*, where each particle has an equal weight. The location of the initial particle set is generated by magnetic localization.

Stage 2: Particle Update. After getting the initialized particle set, the locations of particles at time *t* + 1 are updated by Equation (1).

(1) $$P_{i}^{t+1}=P_{i}^{t}+len_{stp}\left[\begin{array}{c} sin\varphi^{t+1}\\ cos\varphi^{t+1} \end{array}\right]+\lambda^{t+1}$$

where $len_{stp}$, $\varphi^{t+1}$ are the step length and the heading direction at time *t* + 1, respectively. $\lambda^{t+1}$ is the noise following a Gaussian distribution with zero mean and variance 1.

Stage 3: Re-evaluate particle weight. Assuming the magnetic estimation as the system observation, which can be expressed as:

(2) $$M^{t+1}=\left[\begin{array}{c} \begin{array}{c} locx_{M}^{t+1}\\ locy_{M}^{t+1} \end{array}\\ \begin{array}{c} \;len_{stp}\\ \varphi^{t+1} \end{array} \end{array}\right]+\mu^{t+1}$$

where $locx_{M}^{t+1}$, $locy_{M}^{t+1}$ are the location of magnetic estimation at time *t* + 1. $\mu^{t+1}$ is the observation Gaussian noise.

Based on the observation, the particle weight can be re-evaluated as:

(3) $$\omega_{i}^{t+1}=\frac{1}{\sqrt{2\pi \xi }}\exp\left\{-\frac{1}{2\xi }{\left(P_{i}^{t+1}-M^{t+1}\right)}^{T}\left(P_{i}^{t+1}-M^{t+1}\right)\right\}$$

where ξ is the covariance matrix of magnetic estimation.

Stage 4: Normalize particle weights and resampling. The particle weight can be normalized by Equation (4).

(4) $${\bar{\omega }}_{i}^{t+1}=\frac{\omega_{i}^{t+1}}{\sum_{i=1}^{N}\omega_{i}^{t+1}}$$

Afterward, resample the normalized particles, focusing on particles with high weights that are close to the true state, and replicate. We reset the particle weights to ensure that the sum of all weights is 1.

Stage 5: Estimate the fused localization. The fused localization at time *t* + 1 can be estimated by Equation (5).

(5) $$loc^{t+1}=\sum_{i=1}^{N}{\bar{\omega }}_{i}^{t+1}P_{i}^{t+1}$$

Algorithm 1 presents the complete procedures of the proposed fused localization method [47].
**Algorithm 1:** Proposed fused localization method**Input:** The magnetic sequences and IMU sensor data from the smart device.**Output:** The pedestrian location $loc^{t+1}$ at time *t* + 1. 1: Collect data from smart devices. 2: // *Magnetic and PDR-based data pre-processing and localization.* 3: Magnetic data preprocessing are described in Section 3.2. 4: Multi-dimensional dataset as described in Section 3.2. 5: ResNet-GRU-LSTM neural network for magnetic localization, as described in Section 3.3. 6: Multifeature-driven step length estimation as described in Section 3.4. 7: // *Fusion localization by particle filter.* 8: Initialize particle set $P_{i}^{t}=\{\left(locx_{i}^{t},locy_{i}^{t}\right)|i=1,\ldots ,N\}$ at time *t*. 9: **for** each step **do** 10:  **for** each particle **do** 11:    The particle location $P_{i}^{t+1}$ at time *t* + 1 is updated by Equation (1). 12:    The particle weight $\omega_{i}^{t+1}$ is re-evaluated by Equation (3). 13:  **end for** 14:  Normalizing the weights ${\bar{\omega }}_{i}^{t+1}$ by Equation (4). 15:  Resampling the particles. 16:   Estimate the fusion localization $loc^{t+1}$ at time *t* + 1 by Equation (5). 17: **end for**

### 3.2. Data Preprocessing

In this part, we detail the preprocessing of magnetic data and IMU data used for model training.

The geomagnetic field in indoor environments is susceptible to ferromagnetic materials such as iron or steel-containing infrastructure, resulting in unique and distinct ferromagnetic disturbances. Thus, the geomagnetic field could be explored for indoor localization. 

**Figure 2:**
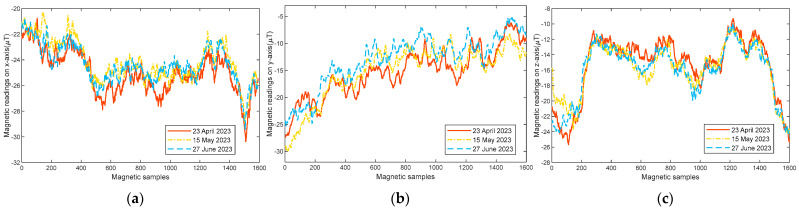
Stability of the magnetic field of the same trajectory collected by a smartphone at different dates: (**a**) magnetic readings on the *x*-axis; (**b**) magnetic readings on the *y*-axis; (**c**) magnetic readings on the *z*-axis.

We conducted extensive experiments to verify the stability of the magnetic field in a 30 m corridor. Figure 2 shows the magnetic field of the same trajectory collected from the smartphone’s three-axis magnetic sensor at different times. It can be seen that although there exist variations in the three-direction magnetic field readings at different dates, the changing trend is strikingly similar. This demonstrates that the magnetic field in indoor environments is quite stable.

**Figure 3:**
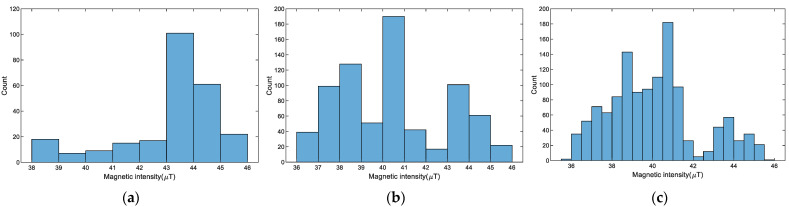
Distribution of magnetic field intensity with various magnetic-containing materials: (**a**) a fire hydrant and two iron doors; (**b**) a fire hydrant, two iron doors, and a metal cabinet; (**c**) two fire hydrants, two iron doors, and two metal cabinets.

Since magnetic uniqueness is caused by ferromagnetic disturbance, the higher the magnetic uniqueness, the better the location discrimination could be. To validate the uniqueness of the magnetic field in indoor environments, we also carried out several experiments to identify the distribution of the magnetic intensity values. As shown in Figure 3, we statistic the magnetic intensity distribution of various magnetic-containing materials in the 30 m corridor. From the figure, the magnetic intensity distribution becomes more diverse as the magnetic-containing materials increase. Besides, the magnetic intensities with similar values are not clustered in a certain range. The experimental results indicate that magnetic field intensity exhibits great uniqueness. 

In a nutshell, the stability and uniqueness of magnetic fields make indoor localization with smartphones highly practical. To improve the discrimination of the magnetic field distribution, more magnetic feature data will be utilized for localization in this paper. Benefiting from the magnetometer sensor built into the smartphone, the three-axis magnetic field can be obtained directly, which is spatial-related and orientation-dependent. Furthermore, richer magnetic feature information can be synthesized by the three-axis magnetic field, like magnetic field intensity and horizontal component. They are relatively stable and orientation-independent of the smartphone, thus efficiently eliminating device heterogeneity. 

Therefore, we construct a magnetic dataset containing 5-dimensional magnetic components, as shown below, which is highly distinguishable and reliable for localization.

(6) $${\tilde{D}}_{mag}=\left[mag_{x},mag_{y},mag_{z},\;mag_{xyz},mag_{h}\right]$$

where $mag_{i},i=x,y,z$ represents the three-axis magnetic field obtained by the magnetometer in a smartphone. $m_{xyz}$, $m_{h}$ represent the magnitude and horizontal component, which can be calculated as Equation (7).

(7) $$\{\begin{array}{c} mag_{xyz}=\sqrt{mag_{x}^{2}+mag_{y}^{2}+mag_{z}^{2}}\\ mag_{h}=\sqrt{mag_{x}^{2}+mag_{y}^{2}} \end{array}$$

The 5-dimensional magnetic dataset needs to be preprocessed before feeding into the neural network model. Since magnetic data collected by different pedestrians on the same experimental path may result in varying geomagnetic densities, we first adopted the resampling method to obtain the same length of magnetic data. To meet the real-time and practical localization requirements, the magnetic dataset is resampled by each step. The step length estimation is determined in Section 3.4, where we put forward a deep learning-based method to achieve accurate step length. 

Then, the median average filtering method with a certain window size is utilized to reduce the noise in the data collection process, which is achieved by removing the maximum and minimum values of the magnetic sequence in the window and then averaging the residual data.

Afterward, to alleviate the effect of magnetic features with varying distributions on the model, we use the Z-score standardization approach to normalize the magnetic dataset, which is formulated by Equation (8).

(8) $$D_{mag}=\frac{{\tilde{D}}_{mag}-\mu_{{\tilde{D}}_{mag}}}{\gamma_{{\tilde{D}}_{mag}}}$$

where $\mu_{{\tilde{D}}_{mag}}$, $\gamma_{{\tilde{D}}_{mag}}$ are the mean and standard deviation of magnetic dataset, respectively. $D_{mag}$ is the normalized magnetic dataset.

In the model training and prediction phases, we utilize a fixed-size sliding window to segment the magnetic sequence of each location label. The proposed ResNet-GRU-LSTM network model provides stronger ability to extract more distinctive features from the magnetic dataset and learn the relations between the magnetic segments and location labels. Thus, pedestrian location based on magnetic data can be achieved. 

**Figure 4:**
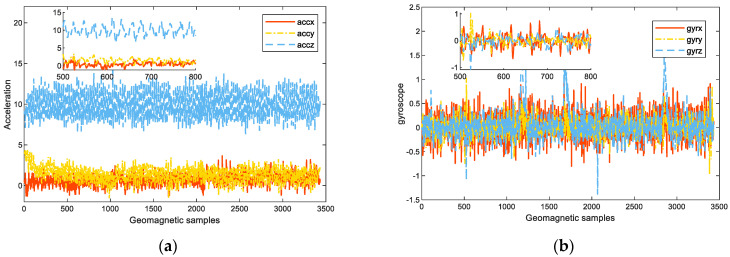
Periodicity of sensor readings: (**a**) acceleration readings; (**b**) gyroscope readings.

The raw inertial data used in this paper contains the three-axis accelerometer and three-axis gyroscope readings, which are collected by a smartphone. As shown in Figure 4, both the acceleration and gyroscope readings change periodically, which is consistent with human walking. Therefore, we can utilize this feature to segment the continuous pedestrian walking process into a single-step accumulation process for step length estimation.

To retain more step length features and make them independent of the smartphone orientation, we also utilized the magnitude of the acceleration and gyroscope to extract features from the temporal aspect, which is determined by the following Equation:

(9) $$\{\begin{array}{c} \left|acc\right|=\sqrt{||acc_{x}||{}_{2}+||acc_{y}||{}_{2}+||acc_{z}||{}_{2}}\\ \left|gyr\right|=\sqrt{||gyr_{x}||{}_{2}+||gyr_{y}||{}_{2}+||gyr_{z}||{}_{2}} \end{array}$$

where $acc_{x}$, $acc_{y}$, $acc_{z}$, $gyr_{x}$, $gyr_{y}$, $gyr_{z}$ are the *x*-, *y*-, *z*-component of the acceleration and gyroscope, respectively.

Thus, the measurements of acceleration and gyroscope can be expressed as below:

(10) $$\{\begin{array}{c} \tilde{acc}=\left[acc_{x},\;acc_{y},\;acc_{z},\;\left|acc\right|\right]\\ \tilde{gyr}=\left[gyr_{x}\;gyr_{y},\;gyr_{z},\;\left|gyr\right|\right] \end{array}$$

An 8-dimensional vector, ${\tilde{D}}_{stp}=\left[\tilde{acc},\;\tilde{gyr}\right]$ is constructed as the step dataset, which is orientation-independent and contains sufficient step information for model training. Before using the step dataset to train the hierarchy GRU neural network, the step dataset needs to be scaled into [0, 1] using MinMaxScaler, which is defined as below:

(11) $$D_{stp}=\frac{{\tilde{D}}_{stp}-\min\left({\tilde{D}}_{stp}\right)}{\max\left({\tilde{D}}_{stp}\right)-\min\left({\tilde{D}}_{stp}\right)}$$

where $\min\left({\tilde{D}}_{stp}\right)$, $\max\left({\tilde{D}}_{stp}\right)$ are the minimum and maximum values of the step dataset.

Afterward, the scaled step dataset will be divided into fragments, with each fragment corresponding to one step. The fragment is generated using a sliding window as follows:

(12) $$f_{i}^{D_{stp}}=\left\{{\tilde{acc}}_{i},\;{\tilde{gyr}}_{i},\;i=1,\ldots ,N\right\}$$

where *i* is the sliding window size, corresponding to the number of acceleration and gyroscope readings in one step.

**Figure 5:**
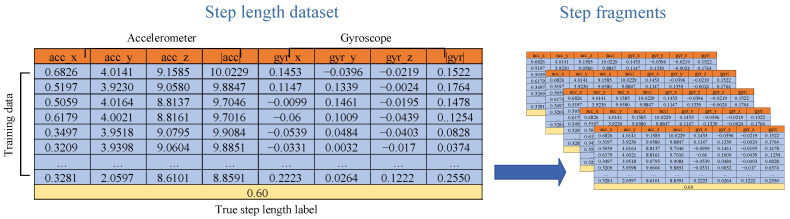
The multi-dimensional step length dataset construction.

Finally, we feed these step fragments and corresponding step length labels to the hierarchy of the GRU neural network for training. The constructed input step dataset with an 8-dimensional feature is shown in Figure 5. 

### 3.3. ResNet-GRU-LSTM Network Model

**Figure 6:**
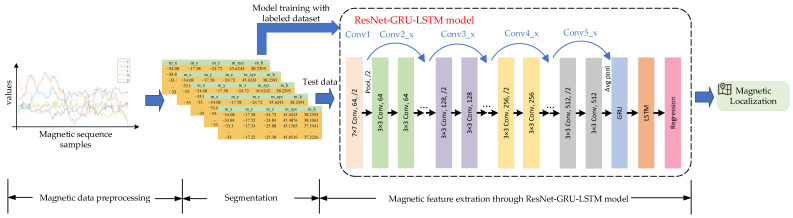
ResNet-GRU-LSTM network model for magnetic localization.

Magnetic signals are highly stable when the indoor environment is unchanged and constant. Abundant magnetic abnormalities contain unique clues that facilitate indoor localization. However, magnetic feature extraction deficiency and similar magnetic signals may exist in complex, symmetric, and large-scale indoor environments, resulting in ambiguous localization during the matching phase. ResNet can effectively capture the local correlated features of the input data, extracting and learning the implied features in the magnetic subsequences. Consider the computational complexity. GRU can reduce computational complexity and further extract the temporal features of the magnetic subsequences. LSTM can extract global temporal features; therefore, accurate predictions can be achieved. Therefore, we propose a ResNet-GRU-LSTM network model for magnetic localization in this paper. The magnetic localization based on the proposed ResNet-GRU-LSTM model is shown in Figure 6. 

In the data preprocessing phase, the magnetic data are collected by smartphones with a pre-installed application. Then, the 5-dimensional magnetic dataset is processed as presented in Section 3.2.

During the data segmentation, a fixed-size sliding window is utilized to segment the magnetic data. Afterward, the segmented data are sent to the ResNet-GRU-LSTM model. The training data with correlation location labels are used to train the model. The test data are used for prediction.

**Figure 7:**
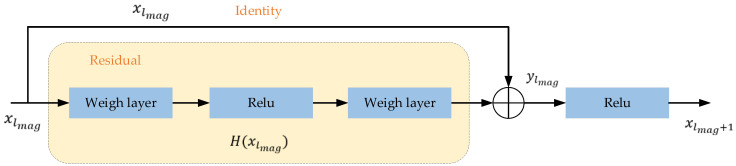
Residual neural network basic module diagram.

In the magnetic feature extraction phase, the multi-dimensional magnetic features will be extracted by ResNet, GRU, and LSTM sequentially. In this paper, ResNet34 [48] is utilized in the neural network model as the first layer, which can solve the degradation problem of neural networks by learning the required mapping using special shortcut connection architectures. Thus, the magnetic features can be efficiently learned and extracted for accurate localization. There are five components in ResNet34: Conv1, Conv2_x, Conv3_x, Conv4_x, and Conv5_x. Conv1 is composed of a convolution layer with a 7×7 convolutional kernel and a max-pooling layer. The remaining components consist of 3, 4, 6, and 3 residual blocks, respectively. Each residual block contains two convolution layers. The residual neural network basic module diagram is shown in Figure 7. The residual block in layer $l_{mag}$ consists of multiple cascaded convolutional layers and a shortcut connection, also called residual mapping and identity mapping. The segmented magnetic sequence $x_{l_{mag}}$ is sent to the ResNet34, but the output of identity mapping is still $x_{l_{mag}}$. The output of residual mapping in layer $l_{mag}$ is defined as:

(13) $$H\left(x_{l_{mag}}\right)=W_{2}\rho \left(W_{1}x_{l_{mag}}\right)$$

where $x_{l_{mag}}$ is the magnetic sequence input of layer $l_{mag}$. $W_{1}$, $W_{2}$ are the weights of each layer. ρ is the ReLu activation function.

Therefore, the general representation of the residual block is shown in Equation (14).

(14) $$\{\begin{array}{c} y_{l_{mag}}=x_{l_{mag}}+H\left(x_{l_{mag}},W_{l_{mag}}\right)\\ x_{l_{mag}+1}=y_{l_{mag}}\rho \end{array}$$

where $x_{l_{mag}+1}$ is the output of layer $l_{mag}$.

By recursion, the magnetic features of multilayer residual mapping at the deep layer $L_{mag}$ can be obtained:

(15) $$x_{L_{mag}}=x_{L_{mag}}+\sum_{i=l_{mag}}^{L_{mag}-1}H\left(x_{i},W_{i}\right)$$

According to the chain rule for derivatives used in backward propagation (BP), the gradient of the loss function *Loss* with respect to $x_{l_{mag}}$ can be expressed in Equation (16).

(16) $$\begin{array}{ll} \frac{\partial Loss}{\partial x_{l_{mag}}} & =\frac{\partial Loss}{\partial x_{L_{mag}}}\cdot \frac{\partial x_{L_{mag}}}{\partial x_{l_{mag}}}\\ & =\frac{\partial Loss}{\partial x_{L_{mag}}}\left(1+\frac{\partial }{\partial x_{mag}}\sum_{i=l_{mag}}^{L_{mag}-1}H\left(x_{i},W_{i}\right)\right)\\ & =\frac{\partial Loss}{\partial x_{L_{mag}}}+\frac{\partial Loss}{\partial x_{L_{mag}}}\cdot \frac{\partial }{\partial x_{l_{mag}}}\sum_{i=l_{mag}}^{L_{mag}-1}H\left(x_{i},W_{i}\right) \end{array}$$

where $\frac{\partial Loss}{\partial x_{L_{mag}}}$ indicates that the gradient of the layer $L_{mag}$ can be passed straightforwardly to any layer $l_{mag}$ shallower than it.

From Equation (16), since $\frac{\partial }{\partial x_{l_{mag}}}\sum_{i=l_{mag}}^{L_{mag}-1}H\left(x_{i},W_{i}\right)$ cannot be −1 all the time during the whole training process, the gradient degradation caused by the network layer will not occur in the residual network.

After extracting the magnetic feature, the output of ResNet will be sent to GRU. As a variant of the LSTM, GRU [49] is relatively simple in construction and consists of two gates, the update gate and the reset gate. Despite the simplicity of the GRU structure, the prediction effect is still superior to that of LSTM, and the training efficiency of the model is also improved.

The update gate is employed to control the ratio of past magnetic information to current information, and the reset gate controls the previous magnetic state information that should be ignored. The equations of GRU with the input magnetic feature data $r^{t}$ at time *t* are defined as follows:

(17) $$\{\begin{array}{c} z_{mag}^{t}=\sigma \left(W_{z_{mag}}\cdot \left[N_{mag}^{t-1},r^{t}\right]+b_{z_{mag}}\right)\\ v_{mag}^{t}=\sigma \left(W_{v_{mag}}\cdot \left[N_{mag}^{t-1},r^{t}\right]+b_{v_{mag}}\right)\\ \begin{array}{c} {\tilde{N}}_{mag}^{t}=\tanh\left(W_{n_{mag}}\cdot \left[v^{t}*N_{mag}^{t-1},r^{t}\right]+b_{n_{mag}}\right)\\ N_{mag}^{t}=\left(1-z_{mag}^{t}\right)*N_{mag}^{t-1}+z_{mag}^{t}*{\tilde{N}}_{mag}^{t} \end{array} \end{array}$$

where $z_{mag}^{t}$, $v_{mag}^{t}$ are the update gate and reset gate. $N_{mag}^{t-1}$ is the neuron output at time *t* − 1. $W_{z_{mag}}$, $W_{v_{mag}}$, $W_{n_{mag}}$ are the weight matrices. $b_{z_{mag}}$, $b_{v_{mag}}$, $b_{n_{mag}}$ denote the deviation vectors. σ is the logistic sigmoid function.

The parameters of GRU need to be updated by backward propagation, which progressively reduces the error by estimating the partial derivatives of the loss function regarding the weights and biases, thus obtaining the error that occurs in each neuron. The order of backpropagation is the opposite of forward propagation. To better understand backward propagation, we redefined some symbols.

The weighted input ($W_{z_{mag}}\cdot \left[N_{mag}^{t-1},r^{t}\right]+b_{z_{mag}}$, $W_{v_{mag}}\cdot \left[N_{mag}^{t-1},r^{t}\right]+b_{v_{mag}}$, $W_{n_{mag}}\cdot \left[v^{t}*N_{mag}^{t-1},r^{t}\right]+b_{n_{mag}}$) of the jth cell at time *t* can be defined as follows:

(18) $$a_{j}^{t}=\underset{i}{\sum }W_{ij}j^{t}$$

The error of the *j*th cell at time *t* is defined as:

(19) $$\delta_{j}^{t}=\frac{\partial Loss}{\partial a_{j}^{t}}$$

where *Loss* is the loss function used to train the model.

Therefore, the gradient of the candidate cell is expressed as:

(20) $$\begin{array}{ll} \delta_{{\tilde{N}}_{mag}}^{t} & =\frac{\partial Loss}{\partial a_{{\tilde{N}}_{mag}}^{t}}=\frac{\partial Loss}{\partial N_{mag}^{t}}\cdot \frac{\partial N_{mag}^{t}}{\partial {\tilde{N}}_{mag}^{t}}\cdot \frac{\partial {\tilde{N}}_{mag}^{t}}{\partial a_{{\tilde{N}}_{mag}}^{t}}\\ & =\frac{\partial Loss}{\partial N_{mag}^{t}}\cdot z_{mag}^{t}\cdot tanh^{’}\left({\tilde{N}}_{mag}^{t}\right) \end{array}$$

The gradient of the reset gate can be determined by Equation (21).

(21) $$\begin{array}{ll} \delta_{v_{mag}}^{t} & =\frac{\partial Loss}{\partial a_{v_{mag}}^{t}}=\frac{\partial Loss}{\partial N_{mag}^{t}}\cdot \frac{\partial Loss}{\partial {\tilde{N}}_{mag}^{t}}\cdot \frac{\partial {\tilde{N}}_{mag}^{t}}{\partial v_{mag}^{t}}\cdot \frac{\partial v_{mag}^{t}}{\partial a_{v_{mag}}^{t}}\\ & =\frac{\partial Loss}{\partial {\tilde{N}}_{mag}^{t}}\cdot \frac{\partial {\tilde{N}}_{mag}^{t}}{\partial a_{{\tilde{N}}_{mag}}^{t}}\cdot \frac{\partial {\tilde{N}}_{mag}^{t}}{\partial v_{mag}^{t}}\cdot \frac{\partial v_{mag}^{t}}{\partial a_{v_{mag}}^{t}}\\ & =\delta_{{\tilde{N}}_{mag}}^{t}\cdot N_{mag}^{t-1}\cdot \sigma^{\prime }\left(a_{v_{mag}}^{t}\right) \end{array}$$

The gradient of the update gate is derived from Equation (22).

(22) $$\begin{array}{ll} \delta_{z_{mag}}^{t} & =\frac{\partial Loss}{\partial a_{z_{mag}}^{t}}\\ & =\frac{\partial Loss}{\partial N_{mag}^{t}}\cdot N_{mag}^{t-1}\cdot \left(-1\right)\cdot \frac{\partial z_{mag}^{t}}{\partial a_{z_{mag}}^{t}}+\frac{\partial Loss}{\partial N_{mag}^{t}}\cdot {\tilde{N}}_{mag}^{t}\cdot \frac{\partial z_{mag}^{t}}{\partial a_{z_{mag}}^{t}}\\ & =\frac{\partial Loss}{\partial N_{mag}^{t}}\cdot \left({\tilde{N}}_{mag}^{t}-N_{mag}^{t-1}\right)\cdot \sigma^{\prime }\left(a_{z_{mag}}^{t}\right) \end{array}$$

Then, the LSTM is utilized for global magnetic feature extraction. LSTM [50] is generally used to process and extract long sequence features with relative intervals and delays in time series. Compared to the recurrent neural network, a long-term memory function unit is added in LSTM, which can solve the gradient vanishing and exploding problems and improve the model prediction ability.

Three gate structures are designed in the basic unit of LSTM: the forgetting gate, input gate, and output gate. The input magnetic feature data $r^{t}$ at time *t*, the cell state $C_{mag}^{t-1}$, and the output of the previous neuron $h_{mag}^{t-1}$ jointly determine the forgetting part of the state memory unit. The formulations of LSTM are presented below:

(23) $$\{\begin{array}{c} i_{mag}^{t}=\sigma \left(W_{i_{mag}}\cdot \left[h_{mag}^{t-1},r^{t}\right]+b_{i_{mag}}\right)\\ f_{mag}^{t}=\sigma \left(W_{f_{mag}}\cdot \left[h_{mag}^{t-1},r^{t}\right]+b_{f_{mag}}\right)\\ \begin{array}{c} o_{mag}^{t}=\sigma \left(W_{o_{mag}}\cdot \left[h_{mag}^{t-1},r^{t}\right]+b_{o_{mag}}\right)\\ \begin{array}{c} C_{mag}^{t}=f_{mag}^{t}*C_{mag}^{t-1}+i_{mag}^{t}*\tanh\left(W_{c_{mag}r}\cdot \left[h_{mag}^{t-1},r^{t}\right]+b_{c_{mag}}\right)\\ h_{mag}^{t}=o_{mag}^{t}tanh\left(C_{mag}^{t}\right) \end{array} \end{array} \end{array}$$

where $i_{mag}^{t}$, $f_{mag}^{t}$, $o_{mag}^{t}$, $C_{mag}^{t}$, and $h_{mag}^{t}$ represent the forget gate, input gate, output gate, memory cell state, and the current neuron outputs, respectively. The subscripts of *W* and *b* denote the weights and biases of the three different gates, i.e., $W_{i_{mag}}$ and $b_{i_{mag}}$ are the weight and bias of input $r^{t}$ at the input gate. tanh is the activation function. σ is the logistic sigmoid function.

The errors in neuron cell output and memory cell state are defined as follows:

(24) $$\{\begin{array}{c} \varepsilon_{h_{mag}}^{t}=\frac{\partial Loss}{\partial h_{mag}^{t}}\\ \varepsilon_{c_{mag}}^{t}=\frac{\partial Loss}{\partial C_{mag}^{t}} \end{array}$$

The gradient of neuron cell output is then calculated by Equations (19) and (24):

(25) $$\begin{array}{ll} \varepsilon_{h_{mag}}^{t} & =\frac{\partial Loss}{\partial h_{mag}^{t}}=\frac{\partial Loss}{\partial a_{j}^{t}}\cdot \frac{\partial a_{j}^{t}}{\partial h_{mag}^{t}}\\ & =\overset{G}{\underset{g=1}{\sum }}\frac{\partial Loss}{\partial a_{g}^{t+1}}\cdot \frac{\partial a_{g}^{t+1}}{\partial h_{mag}^{t}}+\overset{K}{\underset{k=1}{\sum }}\frac{\partial Loss}{\partial a_{k}^{t}}\cdot \frac{\partial a_{k}^{t}}{\partial h_{mag}^{t}}\\ & =\overset{G}{\underset{g=1}{\sum }}\delta_{g}^{t+1}\cdot \frac{\partial a_{g}^{t+1}}{\partial h_{mag}^{t}}+\overset{K}{\underset{k=1}{\sum }}\delta_{k}^{t}\cdot \frac{\partial a_{k}^{t}}{\partial h_{mag}^{t}}\\ & =\overset{G}{\underset{g=1}{\sum }}\delta_{g}^{t+1}\cdot \frac{\partial \left(W_{h_{mag}g}h_{mag}^{t}\right)}{\partial h_{mag}^{t}}+\overset{K}{\underset{k=1}{\sum }}\delta_{k}^{t}\cdot \frac{\partial \left(W_{h_{mag}k}h_{mag}^{t}\right)}{\partial h_{mag}^{t}}\\ & =\overset{G}{\underset{g=1}{\sum }}\delta_{g}^{t+1}\cdot W_{h_{mag}g}+\overset{K}{\underset{k=1}{\sum }}\delta_{k}^{t}\cdot W_{h_{mag}k} \end{array}$$

where *G* represents the number of hidden cell states and *K* represents the number of output layer information.

The gradient of neuron cell output and memory cell state is calculated as follows:

(26) $$\begin{array}{ll} \varepsilon_{c_{mag}}^{t} & =\frac{\partial Loss}{\partial C_{mag}^{t}}=\frac{\partial Loss}{\partial a_{j}^{t+1}}\cdot \frac{\partial a_{j}^{t+1}}{\partial C_{mag}^{t}}+\frac{\partial Loss}{\partial h_{mag}^{t}}\cdot \frac{\partial h_{mag}^{t}}{\partial C_{mag}^{t}}+\frac{\partial Loss}{\partial C_{mag}^{t+1}}\cdot \frac{\partial C_{mag}^{t+1}}{\partial C_{mag}^{t}}\\ & =\delta_{j}^{t+1}\frac{\partial a_{j}^{t+1}}{\partial C_{mag}^{t}}+\varepsilon_{h_{mag}}^{t}\frac{\partial \left[o_{mag}^{t}\tanh\left(C_{mag}^{t}\right)\right]}{\partial C_{mag}^{t}}+\varepsilon_{h_{mag}}^{t}\frac{\partial \left[f_{mag}^{t+1}C_{mag}^{t}+i_{mag}^{t}\sigma \left(C_{mag}^{t+1}\right)\right]}{\partial C^{t}}\\ & =\delta_{f_{mag}}^{t+1}\frac{\partial a_{f_{mag}}^{t+1}}{\partial C_{mag}^{t}}+\delta_{i_{mag}}^{t+1}\frac{\partial a_{i_{mag}}^{t+1}}{\partial C_{mag}^{t}}+\delta_{o_{mag}}^{t+1}\frac{\partial a_{o_{mag}}^{t+1}}{\partial C_{mag}^{t}}+\varepsilon_{h_{mag}}^{t}o_{mag}^{t}tanh^{’}\left(C_{mag}^{t}\right)+\varepsilon_{c_{mag}}^{t+1}f_{mag}^{t+1}\\ & =\varepsilon_{h_{mag}}^{t}o_{mag}^{t}tanh^{’}\left(C_{mag}^{t}\right)+\varepsilon_{c_{mag}}^{t+1}f_{mag}^{t+1}+\delta_{f_{mag}}^{t+1}W_{c_{mag}f_{mag}}+\delta_{i_{mag}}^{t+1}W_{c_{mag}i_{mag}}+\delta_{o_{mag}}^{t+1}W_{c_{mag}o_{mag}} \end{array}$$

Then, according to Equation (19) and $C_{mag}^{t}=f_{mag}^{t}*C_{mag}^{t-1}+i_{mag}^{t}*\tanh\left(W_{c_{mag}r}\cdot \left[h_{mag}^{t-1},r^{t}\right]+b_{c_{mag}}\right)$,

(27) $$\begin{array}{ll} \delta_{c_{mag}}^{t} & =\frac{\partial Loss}{\partial a_{c_{mag}}^{t}}=\frac{\partial Loss}{\partial C_{mag}^{t}}\cdot \frac{\partial C_{mag}^{t}}{\partial a_{c_{mag}}^{t}}\\ & =\varepsilon_{c_{mag}}^{t}\cdot \frac{\partial \left[f_{mag}^{t}C_{mag}^{t-1}+i_{mag}^{t}\sigma \left(a_{c_{mag}}^{t}\right)\right]}{\partial a_{c_{mag}}^{t}}\\ & =\varepsilon_{c_{mag}}^{t}\cdot i^{t}\cdot \sigma^{\prime }\left(a_{c_{mag}}^{t}\right) \end{array}$$

The gradient of the output gate can be obtained by the chain derivative rule according to Equations (19) and $o_{mag}^{t}=\sigma \left(W_{o_{mag}}\cdot \left[h_{mag}^{t-1},r^{t}\right]+b_{o_{mag}}\right)$.

(28) $$\begin{array}{ll} \delta_{o_{mag}}^{t} & =\frac{\partial Loss}{\partial a_{o_{mag}}^{t}}=\frac{\partial Loss}{\partial o_{mag}^{t}}\cdot \frac{\partial o_{mag}^{t}}{\partial a_{o_{mag}}^{t}}=\frac{\partial Loss}{\partial o_{mag}^{t}}\sigma^{\prime }\left(a_{o_{mag}}^{t}\right)\\ & =\frac{\partial Loss}{\partial h_{mag}^{t}}\cdot \frac{\partial h_{mag}^{t}}{\partial o_{mag}^{t}}\cdot \sigma^{\prime }\left(a_{o_{mag}}^{t}\right)=\varepsilon_{h_{mag}}^{t}\cdot \frac{\partial h_{mag}^{t}}{\partial o_{mag}^{t}}\cdot \sigma^{\prime }\left(a_{o_{mag}}^{t}\right)\\ & =\sigma^{\prime }\left(a_{o_{mag}}^{t}\right)\cdot \varepsilon_{h_{mag}}^{t}\cdot \frac{\partial \left[\partial o_{mag}^{t}\tanh\left(C_{mag}^{t}\right)\right]}{\partial o_{mag}^{t}}\\ & =\sigma^{\prime }\left(a_{o_{mag}}^{t}\right)\cdot \varepsilon_{h_{mag}}^{t}\cdot \frac{\partial \left[\partial o_{mag}^{t}\tanh\left(C_{mag}^{t}\right)\right]}{\partial o_{mag}^{t}} \end{array}$$

Afterward, we calculate the gradient of the forget gate, which can be determined by Equation (29).

(29) $$\begin{array}{ll} \delta_{f_{mag}}^{t} & =\frac{\partial Loss}{\partial a_{f_{mag}}^{t}}=\frac{\partial Loss}{\partial f_{mag}^{t}}\cdot \frac{\partial f_{mag}^{t}}{\partial a_{f_{mag}}^{t}}=\frac{\partial Loss}{\partial f_{mag}^{t}}\sigma^{\prime }\left(a_{f_{mag}}^{t}\right)\\ & =\sigma^{\prime }\left(a_{f_{mag}}^{t}\right)\cdot \frac{\partial Loss}{\partial C_{mag}^{t}}\cdot \frac{\partial C_{mag}^{t}}{\partial f_{mag}^{t}}=\sigma^{\prime }\left(a_{f_{mag}}^{t}\right)\cdot \varepsilon_{c_{mag}}^{t}\cdot \frac{\partial C_{mag}^{t}}{\partial f_{mag}^{t}}\\ & =\sigma^{\prime }\left(a_{f_{mag}}^{t}\right)\cdot \varepsilon_{c_{mag}}^{t}\cdot \frac{\partial \left[f_{mag}^{t}C_{mag}^{t-1}+i_{mag}^{t}\sigma \left(a_{c_{mag}}^{t}\right)\right]}{\partial f_{mag}^{t}}\\ & =\sigma^{\prime }\left(a_{f_{mag}}^{t}\right)\cdot \overset{C_{mag}}{\underset{c_{mag}=1}{\sum }}C_{mag}^{t-1}\cdot \varepsilon_{c_{mag}}^{t} \end{array}$$

Finally, the gradient of the input gate is characterized as follows:

(30) $$\begin{array}{ll} \delta_{i_{mag}}^{t} & =\frac{\partial Loss}{\partial a_{i_{mag}}^{t}}=\frac{\partial Loss}{\partial i_{mag}^{t}}\cdot \frac{\partial i_{mag}^{t}}{\partial a_{i_{mag}}^{t}}=\frac{\partial Loss}{\partial i_{mag}^{t}}\sigma^{\prime }\left(a_{i_{mag}}^{t}\right)\\ & =\sigma^{\prime }\left(a_{i_{mag}}^{t}\right)\cdot \frac{\partial Loss}{\partial C_{mag}^{t}}\cdot \frac{\partial C_{mag}^{t}}{\partial i_{mag}^{t}}=\sigma^{\prime }\left(a_{i_{mag}}^{t}\right)\cdot \varepsilon_{c_{mag}}^{t}\cdot \frac{\partial C_{mag}^{t}}{\partial i_{mag}^{t}}\\ & =\sigma^{\prime }\left(a_{i_{mag}}^{t}\right)\cdot \varepsilon_{c_{mag}}^{t}\cdot \frac{\partial \left[f_{mag}^{t}C_{mag}^{t-1}+i_{mag}^{t}\sigma \left(a_{c_{mag}}^{t}\right)\right]}{\partial i_{mag}^{t}}\\ & =\sigma^{\prime }\left(a_{i_{mag}}^{t}\right)\cdot \overset{C_{mag}}{\underset{c_{mag}=1}{\sum }}\sigma \left(a_{c_{mag}}^{t}\right)\cdot \varepsilon_{c_{mag}}^{t} \end{array}$$

After the magnetic feature extraction is completed, the regression layer with two fully connected layers is adopted to output the predicted pedestrian location. Between these layers, the dropout layer is employed to randomly discard a certain number of neurons, which improves the generalization ability of the network and thus prevents overfitting.

In the magnetic localization phase, the predicted localization can be obtained by inputting the test data into the pre-trained ResNet-GRU-LSTM model.

**Figure 8:**
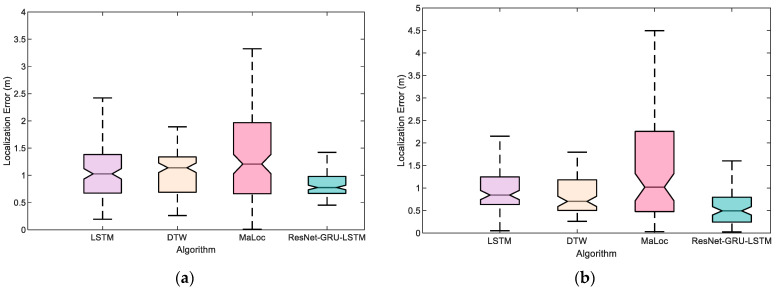
The proposed ResNet-GRU-LSTM model results with two different pedestrians in the experimental scene: (**a**) Pedestrian #1. (**b**) Pedestrian #2.

To evaluate the proposed ResNet-GRU-LSTM localization performance, we conducted experiments on a 90 m path. Two pedestrians (#1, #2) with different heights are invited to collect magnetic test sequences. Figure 8 shows the localization performance with different pedestrians at two trial sites. It can be seen that there is a significant improvement when using our proposed ResNet-GRU-LSTM model. The median and maximum quartiles are smaller than those of the LSTM, DTW, and MaLoc models. This could be attributed to the fact that the proposed ResNet-GRU-LSTM model can extract more features than other magnetic state-of-the-art models. 

### 3.4. Multifeatured-Driven Step Length Estimation

**Figure 9:**
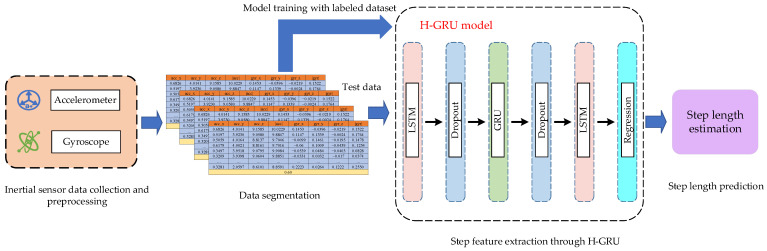
Multifeatured-driven step length estimation.

A nonlinear model is usually used to estimate step length, which can predict accurate step lengths closer to the real state of the pedestrian. A deep neural network is a nonlinear model that utilizes multiple nonlinear transformations to extract and learn high-level abstract features of step length [51]. LSTM can selectively extract the implied antecedent information from the input inertial data, thus retaining the rich local correlation information. GRU can reduce computational complexity and further extract and explore the features of inertial data. Therefore, we propose a hierarchy GRU neural network (H-GRU) model for step length estimation that can extract unique features effectively for accurate step length estimation. Figure 9 depicts the proposed multifeatured-driven step length estimation method. Four parts are included in this method. 

In the data collection and preprocessing phase, the three-axis accelerometer and three-axis gyroscope data are collected by a smartphone. The construction of the 8-dimensional step data set is described in Section 3.2.

In the data segmentation phase, the processed step dataset will be divided into fragments using a sliding window, with each fragment corresponding to one step. 

In the step feature extraction phase, the segmented step dataset is sent to the H-GRU model. LSTM is adopted to extract implied features from the step dataset; more related information will be retained. The equations of LSTM with the input step dataset $D_{stp}^{t}$ at time *t* are defined as follows:

(31) $$\{\begin{array}{c} i_{stp}^{t}=\sigma \left(W_{i_{stp}}\cdot \left[h_{stp}^{t-1},D_{stp}^{t}\right]+b_{i_{stp}}\right)\\ f_{stp}^{t}=\sigma \left(W_{f_{stp}}\cdot \left[h_{stp}^{t-1},D_{stp}^{t}\right]+b_{f_{stp}}\right)\\ \begin{array}{c} o_{stp}^{t}=\sigma \left(W_{o_{stp}}\cdot \left[h_{stp}^{t-1},D_{stp}^{t}\right]+b_{o_{stp}}\right)\\ C_{stp}^{t}=f_{stp}^{t}*C_{stp}^{t-1}+i_{stp}^{t}*\tanh\left(W_{c_{stp}r}\cdot \left[h_{stp}^{t-1},D_{stp}^{t}\right]+b_{c_{stp}}\right)\\ h_{stp}^{t}=o_{stp}^{t}tanh\left(C_{stp}^{t}\right) \end{array} \end{array}$$

where $i_{stp}^{t}$, $f_{stp}^{t}$, $o_{stp}^{t}$, $C_{stp}^{t}$, and $h_{stp}^{t}$ represent the forget gate, input gate, output gate, memory cell state, and the current neuron outputs, respectively.

Then, the extracted features are sent to GRU. The equations of GRU are shown below:

(32) $$\{\begin{array}{c} z_{stp}^{t}=\sigma \left(W_{z_{stp}}\cdot \left[N_{stp}^{t-1},D_{stp}^{t}\right]+b_{z_{stp}}\right)\\ v_{stp}^{t}=\sigma \left(W_{v_{stp}}\cdot \left[N_{stp}^{t-1},D_{stp}^{t}\right]+b_{v_{stp}}\right)\\ \begin{array}{c} {\tilde{N}}_{stp}^{t}=\tanh\left(W_{n_{stp}}\cdot \left[v^{t}*N_{stp}^{t-1},D_{stp}^{t}\right]+b_{n_{stp}}\right)\\ N_{stp}^{t}=\left(1-z_{stp}^{t}\right)*N_{stp}^{t-1}+z_{stp}^{t}*{\tilde{N}}_{stp}^{t} \end{array} \end{array}$$

where $z_{stp}^{t}$, $v_{stp}^{t}$ are the update gate and reset gate. $N_{stp}^{t-1}$ is the neuron output at time *t-1*. The backward propagation is presented in Section 3.3.

Then, the extracted features will be passed to the last LSTM layer to extract global time-series information. Finally, these well-extracted features are randomly dropped and fed to the regression layer. To prevent overfitting, each layer is followed by a dropout layer with a dropout rate of 0.2. The regression layer contains two fully connected layers with the sigmoid activation function. The regression layer maps the feature vectors of the inertial data sequence to the correlating pedestrian step length, thus establishing a mapping relationship between the inertial data and the step length.

Therefore, the proposed multifeatured-driven step length estimation can be characterized as:

(33) $$stp_{pre}=Net_{H-GRU}\left(f_{i}^{D_{stp}}\right)$$

where $f_{i}^{D_{stp}}$ is the segmented test step datasets, which are calculated in Section 3.2. $Net_{H-GRU}$ is the proposed H-GRU neural network.

**Figure 10:**
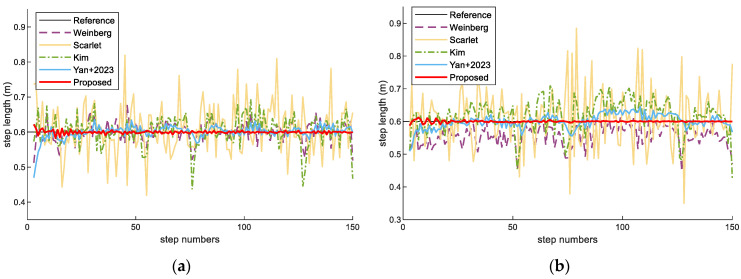
Step length estimation results with two pedestrians in the experimental scenes: (**a**) Pedestrian #1; (**b**) Pedestrian #2.

We have compared the proposed multifeatured-driven step length estimation with Weinberg [52], Scarlet [53], Kim [54], and a method related to our previous work [25] in the 90 m path, as shown in Figure 10. In this paper, the pedestrians walk at a constant pace of 0.6 m per step. From the results, our proposed method can estimate a more precise and reliable step length for different-height pedestrians. This is mainly because deep learning-based step length estimation can learn more accurate features to estimate the step length per stride. 

## 4. Experimental Results and Evaluation

In this section, we first present the details of the experimental setup in Section 4.1. Afterward, the step length performance and localization performance are analyzed in Section 4.2 and Section 4.3. Finally, the time overhead is presented in Section 4.4.

**Figure 11:**
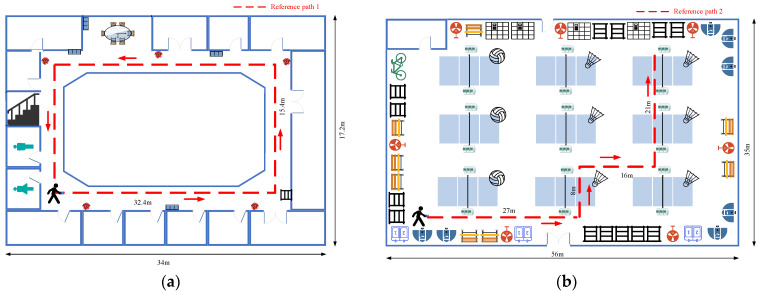
Layout plans of the experimental scenes: (**a**) Scene 1; (**b**) Scene 2.

Extensive experiments are conducted at two different indoor trial sites to authenticate the performance of the proposed localization system. As shown in Figure 11, the first site is a rectangular symmetric corridor outside the office area covering 34 × 17.2 × 5 $m^{3}$, and the second site is an open area in the school gym covering 56 × 35 × 12 $m^{3}$. Two typical experimental trajectories are devised: reference path 1 and reference path 2. The movement trajectory follows the red solid arrow. Reference path 1 is a 92 m rectangular path in a narrow corridor, containing several fire hydrants, iron cabinets, and iron railings. There are also some iron doors along the path. Since the width of the path is narrow and surrounded by many iron-containing materials, the magnetic anomalies in reference path 1 are quite abundant. Reference path 2 is a 72 m continuous, curved path in an open area. Some sections of the path contain iron and ferrous substances, such as iron chairs, basketball stands, electric fans, and iron bars. Other sections are not surrounded by ferrous materials, resulting in weak magnetic anomalies. Therefore, these experiment scenes are LOS (line of sight) and non-enclosed scenarios without large obstacles.

### 4.1. Experimental Setup

Application terminal: The application is developed based on the Android operating system and then pre-installed on smartphones. When the pedestrian holds the smartphone while walking along the reference paths, the accelerometer, gyroscope, and magnetometer data will be collected at the same time. During the data collection phase, the sampling frequency is 50 Hz. 

Server terminal: TensorFlow 2.9.0 is utilized as the machine learning framework to build our model, which is running under the Windows 10 64-bit operating system. The Windows operating system has an Intel i7-9700 CPU and a P620 GPU. The rest of the system configuration is listed in Table 1.

According to the terrain layout, we plan several training data collection paths near the reference path with the same distance interval. The pedestrians are required to walk along these planned paths in the heading direction with a smartphone. The accelerometer, gyroscope, and magnetometer training data will be collected at a 50 Hz sampling frequency. To better learn the contextual relationships between training datasets and labels, we utilize a sliding window to segment training datasets during the training process. The form of the dataset used in this paper is introduced below.

Magnetic fingerprint datasets: In the magnetic-based localization, the training datasets ${\tilde{D}}_{mag}$ mainly contain 5-dimensional fingerprint components $\left[mag_{x},mag_{y},mag_{z},\;mag_{xyz},mag_{h}\right]$ and corresponding location labels. At trial site 1, each dataset is in the form of 20 × 4716. At trial site 2, each dataset is in the form of 20 × 2756. After segmentation by sliding window, 131 and 85 fragments with a size of 20 × 180 × 5 were obtained, respectively.

Step length datasets: In the step length estimation, the training datasets contain accelerometer, gyroscope data, ${\tilde{D}}_{\mathrm{stp}}=\left[\tilde{acc},\;\tilde{gyr}\right]$, and corresponding step length labels. At trial site 1, the size of the training dataset is 3433 × 8. At trial site 2, the size of the training dataset is 2755 × 8. The segments are 150 and 110, with a size of 1 × 130 × 8, respectively.

Hyperparameter Setting: To prevent too many variables from causing inaccurate experimental results, we set the hyperparameters of deep neural networks basically the same. Table 2 shows the setting of hyperparameters for deep networks in this paper. It should be noted that in the following analysis, if not specifically mentioned, the values of these hyperparameters remain unchanged.

We recruited two pedestrians for the follow-up experiments, using four different mobile devices to collect accelerometer, gyroscope, and magnetometer test data. Pedestrian (#1) is a female with 164 cm, and pedestrian (#2) is a male with 183 cm. The mobile devices are the Vivo X30 (Guangdong, China), Huawei Mate30 (Guangdong, China), Vivo Y85a (Guangdong, China), and Xiaomi 10 (Beijing, China). We present the technical parameters of these smartphones in Table 3. At the trial sites, pedestrians were asked to walk through the reference paths several times.

In this paper, landmarks are set every 0.6 m along the planned paths, which is the ground truth location. The pedestrian holds the smartphone while walking along these landmarks; the data are collected automatically. In the localization performance evaluation phase, the predicted locations of the pedestrians can be obtained by our proposed system. Therefore, the accuracy of the proposed system can be achieved by comparing the predicted locations with the ground-truth locations of each step. 

### 4.2. Step Length Performance

**Figure 12:**
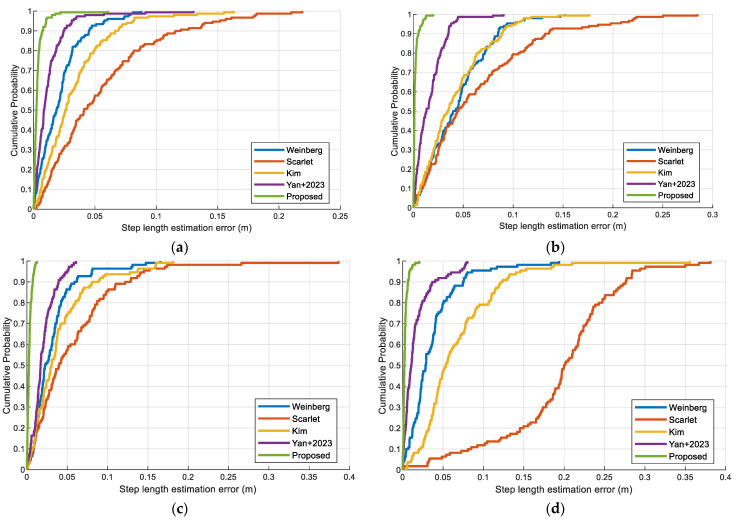
The errors of the proposed step length estimation and other methods: (**a**) Pedestrian #1 in scene 1. (**b**) Pedestrian #2 in scene 1. (**c**) Pedestrian #1 in scene 2. (**d**) Pedestrian #2 in scene 2.

To evaluate the proposed multifeatured-driven step length estimation, we compared the errors of these step estimation methods as presented in Figure 12. These experiments demonstrate that the proposed step length estimation method can achieve the smallest error compared to other step length estimation methods. Regardless of the different pedestrians or scenarios, the proposed step length estimation can achieve better performance. 

**Figure 13:**
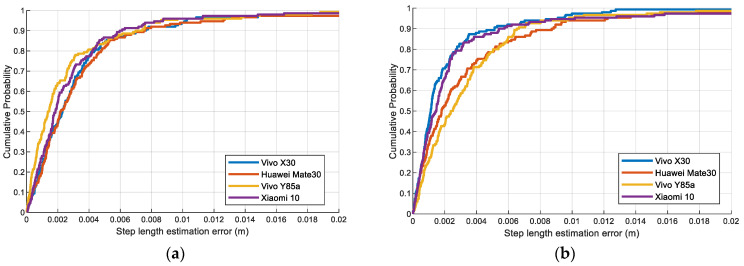

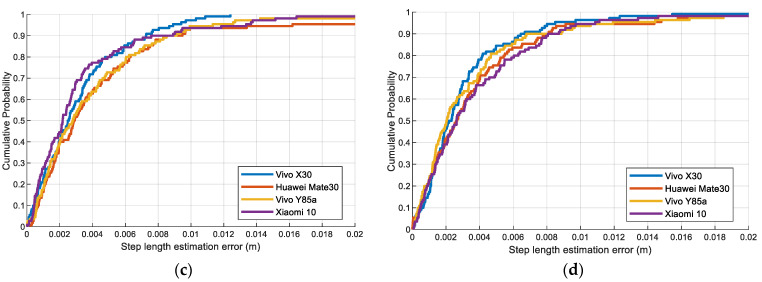
The CDF of step length estimation errors of the proposed method using four different devices: (**a**) Pedestrian #1 in scene 1. (**b**) Pedestrian #2 in scene 1. (**c**) Pedestrian #1 in scene 2. (**d**) Pedestrian #2 in scene 2.

Figure 13 depicts the CDF of step length estimation error with different devices, pedestrians, and trial sites to verify its practicality. Vivo X30, Huawei Mate30, Vivo Y85a, and Xiaomi 10 are used in the experiment. A similar trend of the curves is observed in these figures, which demonstrate the robustness and feasibility of the proposed step length method. This is because accurate step length features can be extracted by our proposed step length method, which helps overcome device heterogeneity. 

**Figure 14:**
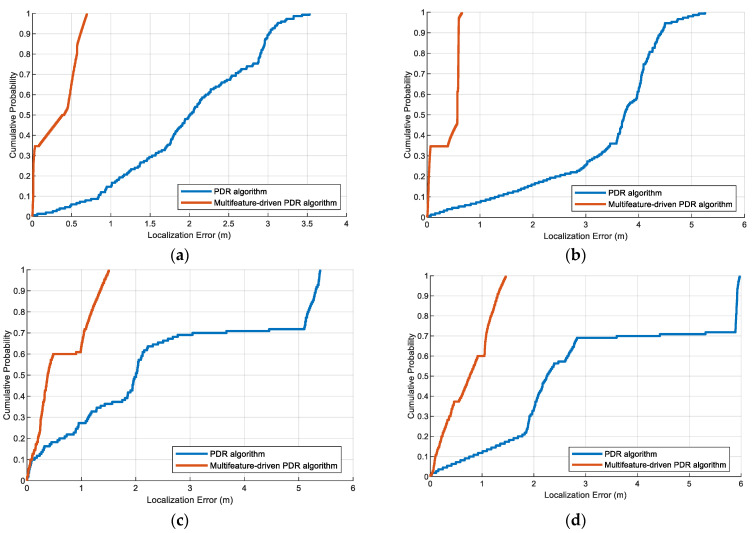
The localization errors of multifeatured-driven PDR and PDR methods in the experimental scenes: (**a**) Pedestrian #1 in scene 1. (**b**) Pedestrian #2 in scene 1. (**c**) Pedestrian #1 in scene 2. (**d**) Pedestrian #2 in scene 2.

We have conducted some experiments to show the localization errors of the multifeatured-driven PDR and traditional PDR methods. The results are illustrated in Figure 14. From these figures, it can be seen that the multifeatured-driven PDR achieves better accuracy and performance than the traditional PDR algorithm. This is achieved because our step length estimation can predict an accurate step length based on extracted features, which improves the localization estimation. 

### 4.3. Localization Performance

To validate the performance of the proposed fusion localization algorithm, we compare our proposed method with LSTM, GRU, PDR, DTW, and MaLoc methods in several aspects. Two different indoor scenes and two pedestrians (#1, #2) with different heights are involved in the experiments. 

The LSTM and GRU neural networks are used in the LSTM and GRU localization, respectively. The PDR method is achieved by typical step length and heading angle estimation. The DTW algorithm calculates and matches the maximum magnetic similarity between online magnetic data and a fingerprint database to predict the location. The MaLoc algorithm utilizes a particle filter to achieve localization. 

**Figure 15:**
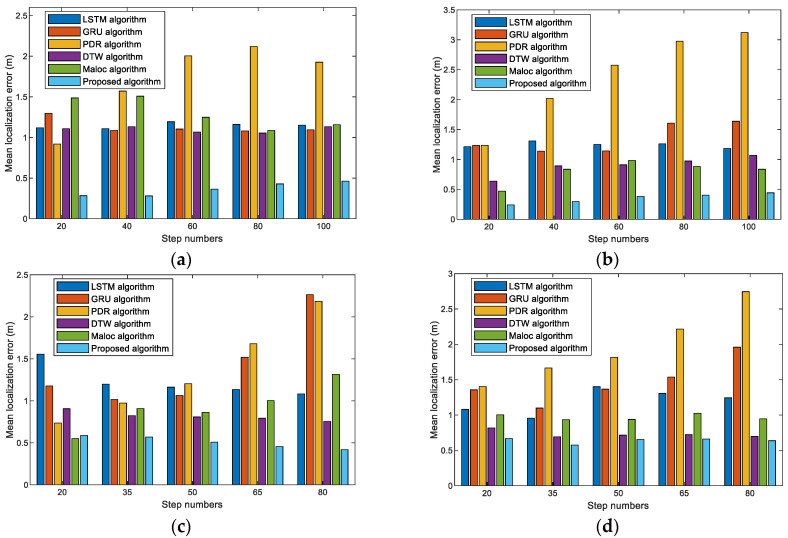
Mean localization errors of LSTM, GRU, PDR, DTW, MaLoc, and the proposed method under varied step numbers: (**a**) Pedestrian #1 in scene 1. (**b**) Pedestrian #2 in scene 1. (**c**) Pedestrian #1 in scene 2. (**d**) Pedestrian #2 in scene 2.

Figure 15 presents the schematic diagram of the mean localization errors for LSTM, GRU, PDR, DTW, MaLoc, and the proposed method with various pedestrians, sites, and steps. These experimental results demonstrate that our method achieves a lower mean error in comparison to other methods. The mean error of our proposed method remains essentially stable as the step number increases. The proposed method achieves comparable accuracy for two reasons, as follows: First, the ResNet-GRU-LSTM neural network model has a better ability to capture the relationships between magnetic features and location labels than single neural network models, thus achieving more accurate prediction. Second, the hybrid neural network model for step length estimation can compute step lengths that are close to the true step length, which can effectively solve the problem of the cumulative errors caused by pedestrian dead reckoning. 

**Figure 16:**
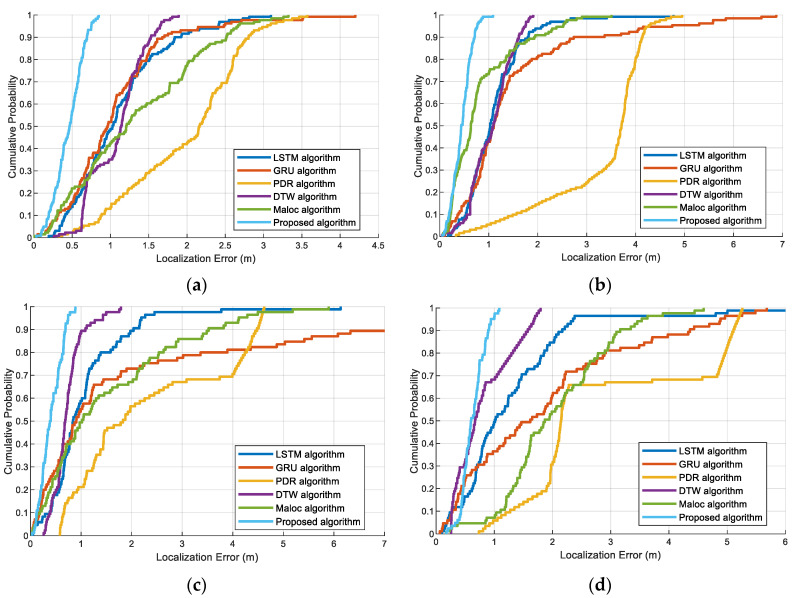
The CDF of localization error with LSTM, GRU, PDR, DTW, MaLoc, and the proposed method at two experiment sites: (**a**) Pedestrian #1 in scene 1. (**b**) Pedestrian #2 in scene 1. (**c**) Pedestrian #1 in scene 2. (**d**) Pedestrian #2 in scene 2.

The CDF of localization error for different methods is shown in Figure 16. From these figures, it can be seen that the localization error of our proposed method is the smallest compared to those of state-of-the-art methods in different scenarios with different pedestrians. These results occur because the ResNet exhibits an outstanding ability for feature extraction, and the LSTM could extract global temporal magnetic features for more accurate information; thus, the forecasting ability of the ResNet-GRU-LSTM model is further enhanced. Besides, the localization algorithm fused with the deep learning-based PDR algorithm can effectively eliminate the ambiguous location errors caused by magnetic and PDR localization. 

**Figure 17:**
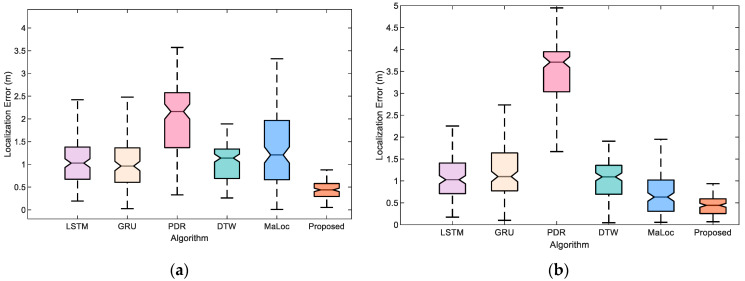

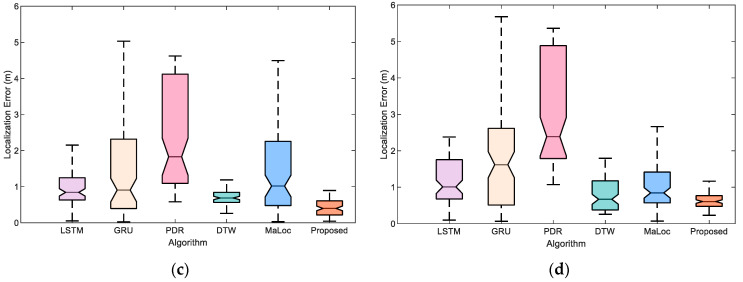
Localization error with LSTM, GRU, PDR, DTW, MaLoc, and the proposed fusion method at two experiment scenes: (**a**) Pedestrian #1 in scene 1. (**b**) Pedestrian #2 in scene 1. (**c**) Pedestrian #1 in scene 2. (**d**) Pedestrian #2 in scene 2.

Figure 17 clearly illustrates the localization errors of different algorithms in two scenes. As can be seen from the results, our proposed fusion method exhibits great localization performance and has fewer outliers than the LSTM, GRU, PDR, DTW, and MaLoc methods. This is because our proposed fusion method can extract sufficient magnetic and step length information to achieve accurate localization with different scenarios and pedestrians, thus removing the anomalies. 

**Figure 18:**
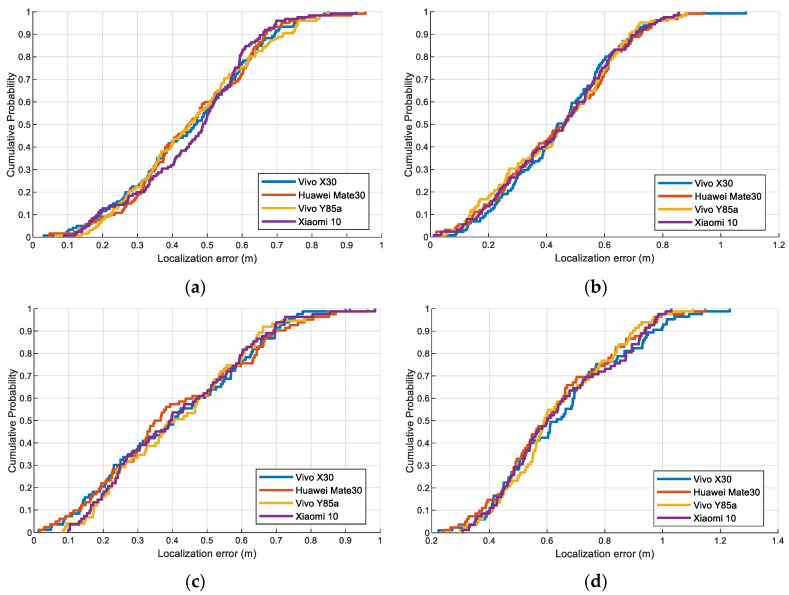
The CDF of the proposed localization method using Vivo X30, Huawei Mate30, Vivo Y85a, and Xiaomi 10 devices: (**a**) Pedestrian #1 in scene 1. (**b**) Pedestrian #2 in scene 1. (**c**) Pedestrian #1 in scene 2. (**d**) Pedestrian #2 in scene 2.

We also conducted experiments to examine the localization error with two pedestrians using four different devices, as shown in Figure 18. All these experimental results show that these curves have similar tendencies and proximity, which demonstrate the good robustness and practicality of our proposed model, achieving promising performance in different scenarios and equipment. This is attributed to the fact that the input data of both neural network models are multidimensional sequences that contain spatial and temporal information. Therefore, our method can obtain sufficient features for localization and avoid errors caused by heterogeneous equipment. 

Table 4 and Table 5 present the 75th and 95th percentile localization errors with two pedestrians (#1, #2) in two experimental sites. A significant improvement in the localization accuracy of our proposed method can be seen from the tables, which achieve considerable accuracy compared to other state-of-the-art algorithms. This is mainly because our method can obtain more location features from the neural network models to achieve localization more accurately than other methods, thus avoiding anomalies generated by magnetic and PDR localization. 

The mean errors and root mean square error (RMSE) of LSTM, GRU, PDR, DTW, MaLoc, and the proposed method with different pedestrians (#1, #2) and sites are presented in Table 6 and Table 7. The results obtained so far show that significant improvement has been achieved by our proposed method for different pedestrians and scenarios. This is due to the fact that the proposed method can efficiently eliminate the errors and outliers generated during the localization process; thus, the localization performance is markedly enhanced. 

### 4.4. Overhead

Additionally, we present a comparison of the computational complexity of our proposed algorithm with other algorithms for pedestrians (#1, #2) at scenes 1 and 2, as shown in Table 8. It should be noted that the model training time is not counted; only the testing time is included. From the table, it can be seen that LSTM, GRU, and PDR methods require less time than our method, while DTW and MaLoc take more time to achieve localization. The time required to predict localization by our proposed method does not exceed 15 s, which means that the computational complexity of our algorithm is comparatively low. Considering its accuracy, the localization system proposed in this paper has good localization performance and good generality and feasibility in indoor localization. 

## 5. Conclusions

In this paper, we propose a deep neural network-based fusion indoor localization system with smartphones that combines magnetic estimation and PDR under a particle filter framework. To improve feature extraction ability and localization accuracy, we propose a ResNet-GRU-LSTM network model for magnetic localization. Afterward, a multifeatured-driven step length estimation method is proposed, using a hierarchy GRU neural network to extract more step information from an 8-dimensional dataset for more accurate step length estimation. Thus, multifeatured-driven PDR localization with higher accuracy can be achieved than traditional PDR localization. Finally, we utilize a particle filter to integrate magnetic and multifeatured-driven PDR localization for more reliable and robust localization results. 

We have conducted extensive experiments at two different indoor sites, which are a 584.8 $m^{2}$ office area and a 1960 $m^{2}$ school gym. Two pedestrians with Vivo X30, Huawei Mate30, Vivo Y85a, and Xiaomi 10 smartphones are involved in the experiments. Based on the localization performance results of ResNet-GRU-LSTM, this neural network model can achieve the best localization accuracy among other magnetic state-of-the-art models. The ability of magnetic feature extraction is effectively improved, regardless of the different pedestrians or scenarios. For the multifeatured-driven step length estimation algorithm, our method can predict more accurate step length compared with other step estimation methods, especially in long-distance localization. Therefore, the localization performance of PDR with multifeatured-driven step length estimation is also greatly improved. As can be seen from the proposed fusion localization system results, it shows that our proposed localization system has more stable and reliable localization performance compared with other state-of-the-art methods, for which higher accuracy can be achieved. Meanwhile, our proposed fusion localization can effectively mitigate the equipment heterogeneity and achieve great robustness. Moreover, the computational complexity of our proposed fusion localization is relatively small. Therefore, the proposed fusion localization system is feasible for indoor LBS, which has vast application prospects in different scenarios, pedestrians, and smartphones. 

In this paper, although these experiments are conducted with four different smartphones, the pedestrians are holding smartphones in a fixed attitude to collect data. Different holding postures will affect localization accuracy. In the future, we will consider using deep learning methods to address this issue. In addition, long-distance localization in more complex environments will be explored to test our proposed fusion localization system. Another future research direction is 3D location-based services, which can provide accurate floor location when pedestrians are in a high-rise building. 

## Figures and Tables

**Table 1:** System configuration of the server terminal.

Category	Description
CPU	Intel i7-9700 CPU @ 3.00 GHz
GPU	P620
RAM	8 GB
Operation system	Windows 10 64-bit
Language	Python 3.10
Framework	TensorFlow 2.9.0
Library	Keras 2.9.0

**Table 2:** Hyperparameters for deep networks.

Hyperparameters	Setting
Input dimension	20 × 180 × 5/1 × 130 × 8
Hidden layers	3
Number of hidden units	100
Drop probability between each layer	0.2
Number of epochs	10
Learning rate	5 × 10^−3^
Optimizer	Adam
Loss function	Mse
Activation function	Sigmoid

**Table 3:** The technical parameters of the smartphones.

Technical Parameters	Vivo X30	Huawei Mate30	Vivo Y85a	Xiaomi 10
Operation system	Android 9	Android 10	Android 8	Android 10
Image resolution	2400 × 1080	2340 × 1080	2280 × 1080	2340 × 1080
CPU	Exynos 980	Snapdragon 990	Snapdragon 450	Snapdragon 865
RAM and ROM	8 G + 256 G	8 G + 128 G	4 G + 64 G	8 G + 128 G
Battery capacity	4350 mAh	4100 mAh	3260 mAh	4780 mAh
Screen	6.44 inch	6.62 inch	6.26 inch	6.67 inch

**Table 4:** The 75th and 95th percentiles localization errors of LSTM, GRU, PDR, DTW, MaLoc, and the proposed methods with two pedestrians (#1, #2) in scene 1.

	Method	75th Percentile (m)	95th Percentile (m)
Scene 1 (Pedestrian #1)	LSTM	1.3807	2.4115
	GRU	1.3625	2.4639
	PDR	2.5758	3.0459
	DTW	1.3371	1.6481
	MaLoc	1.9665	2.6639
	Proposed	0.5928	0.7508
Scene 1 (Pedestrian #2)	LSTM	1.4075	2.0970
	GRU	1.6376	4.7171
	PDR	3.9488	4.2651
	DTW	1.3554	1.7493
	MaLoc	1.0205	2.5128
	Proposed	0.5778	0.7659

**Table 5:** The 75th and 95th percentiles localization errors of LSTM, GRU, PDR, DTW, MaLoc, and the proposed methods with two pedestrians (#1, #2) in scene 2.

	Method	75th Percentile (m)	95th Percentile (m)
Scene 2 (Pedestrian #1)	LSTM	1.2450	2.2110
	GRU	2.3167	9.3482
	PDR	4.1186	4.5615
	DTW	0.8428	1.4330
	MaLoc	2.2558	4.1609
	Proposed	0.5774	0.7446
Scene 2 (Pedestrian #2)	LSTM	1.7548	2.3577
	GRU	2.6148	4.9046
	PDR	4.8981	5.1876
	DTW	1.1743	1.6870
	MaLoc	1.4148	2.2267
	Proposed	0.7439	0.9644

**Table 6:** The mean and RMS errors of LSTM, GRU, PDR, DTW, MaLoc, and the proposed methods with two pedestrians (#1, #2) in scene 1.

	Method	Mean Error (m)	RMS Error (m)
Scene 1 (Pedestrian #1)	LSTM	1.1035	1.2507
	GRU	1.0496	1.2456
	PDR	1.9790	2.1154
	DTW	1.0824	1.1381
	MaLoc	1.3190	1.5503
	Proposed	0.4522	0.4892
Scene 1 (Pedestrian #2)	LSTM	1.1138	1.2719
	GRU	1.4794	1.9811
	PDR	3.3116	3.4653
	DTW	1.0540	1.1330
	MaLoc	0.8365	1.0913
	Proposed	0.4492	0.4899

**Table 7:** The mean and RMS errors of LSTM, GRU, PDR, DTW, MaLoc, and the proposed methods with two pedestrians (#1, #2) in scene 2.

	Method	Mean Error (m)	RMS Error (m)
Scene 2 (Pedestrian #1)	LSTM	1.0693	1.3613
	GRU	2.1594	3.5729
	PDR	2.3258	2.7509
	DTW	0.7294	0.7887
	MaLoc	1.5126	2.0207
	Proposed	0.3927	0.4520
Scene 2 (Pedestrian #2)	LSTM	1.2848	1.7088
	GRU	1.8603	2.3792
	PDR	2.8906	3.2625
	DTW	0.7995	0.9272
	MaLoc	2.0418	2.2314
	Proposed	0.6326	0.6616

**Table 8:** The computational complexity of LSTM, GRU, PDR, DTW, MaLoc, and the proposed methods with two pedestrians (#1, #2) in two experimental scenes.

	Method	Time Overhead (#1) (s)	Time Overhead (#2) (s)
Scene 1	LSTM	6.83	7.10
	GRU	6.94	6.87
	PDR	5.21	4.88
	DTW	1584.28	1452.2
	MaLoc	31.41	30.13
	Proposed	14.67	14.09
Scene 2	LSTM	4.74	4.69
	GRU	4.70	4.88
	PDR	1.73	1.17
	DTW	395.38	400.89
	MaLoc	32.39	29.38
	Proposed	9.69	9.39

## Data Availability

All test data mentioned in this paper will be made available on request to the corresponding author’s email with appropriate justification.
